# Provision of a protein-rich supplement for grazing suckling female beef calves to improve productive performance and metabolic response

**DOI:** 10.5713/ab.21.0226

**Published:** 2021-10-29

**Authors:** Deilen S Moreno, Román M Ortega, David C Marquez, Thiago R Moreira, Edson J dos Santos, Daniel M de Almeida, Mário F Paulino, Luciana N Rennó, Edenio Detmann

**Affiliations:** 1 Faculty of Agricultural Sciences, University of Pamplona, Pamplona, 543050, Colombia; 2 Faculty of Agricultural Science, University of Cundinamarca, Fusagasugá, 252211, Colombia; 3 Animal Science Department, Federal University of Viçosa, Viçosa, 36570-900, Brazil

**Keywords:** Biopsy, Cattle, Gene Expression, Gluconeogenesis, Ruminant Feeding

## Abstract

**Objective:**

This study was conducted to evaluate the effects of the provision of a protein-rich supplement on productive performance, and metabolic profile on grazing suckling female beef calves in tropical conditions during 150 d of experimentation.

**Methods:**

Fifty-six Nellore suckling female calves, and their respective dams were distributed in a completely randomised design and made to undergo two treatments as follows: UNS (without supplementation), and SUP (supplementation with 5 g/kg body weight [BW] of a protein supplement). Throughout the experiment, animal performance and metabolic profile were evaluated. Also, ureagenesis and gluconeogenesis were assessed for gene expression.

**Results:**

SUP female calves showed a higher voluntary intake (p≤0.03) of the diet components evaluated, digestibility of organic matter (p≤0.02) and microbial nitrogen production (MICN; p≤0.02) compared to UNS female calves. In its turn, serum urea nitrogen (p≤0.01) and insulin-like growth factor-1 (p≤0.03) levels and ureagenesis (p≤0.04) increased in SUP female calves compared to UNS female calves. Blood glucose and triglyceride levels were not affected by supplementation. The average daily gain (ADG) from SUP female calves was higher (p≤0.02) compared with UNS female calves. However, supplementation did not affect the body measures of the animals.

**Conclusion:**

In summary, provision of a protein-rich supplement improves the intake and nutrients digestibility, ADG and final BW and increases metabolic indicators of the protein status in grazing suckling female beef calves in tropical conditions.

## INTRODUCTION

The performance of grazing suckling calves mainly depends on the intake of nutrients from maternal milk, which is not sufficient to meet their requirements after 3 mo of age, to support potential growth [1]. Therefore, calves rely increasingly on nutrient intake from pasture during this time. However, simultaneously, with the post-peak decline of the dam lactation curve [2], there is a decrease in pasture nutritive value and forage mass due to the transition from the rainy to dry season in Brazilian conditions. This scenario can lead to nutrient imbalances or limitations that compromise forage intake and digestion or metabolism of absorbed substrates [3] at a time when protein and energy requirements of calves increase as growth progresses. As a consequence, lower gain rates are observed in the animals, suggesting that supplemental nutrients may be required [2].

In this sense, offering a protein-rich supplements in the creep-feeding system for grazing suckling calves is a strategy used to increase nutrient intake, optimise weight gain, and can lead to metabolic changes in indicators of protein statuses, such as glucose, triglycerides, total proteins, albumin, and urea in the tropics.

On the other hand, many studies evaluating provision of different supplements on changes in the expression of some genes associated with gluconeogenesis (phosphoenolpyruvate carboxykinase [PEPCK]) and ureagenesis (carbamoyl phosphate synthetase-1 [CPS-1]) hepatic in cattle have been performed. Some authors have reported an increase in hepatic mRNA expression of PEPCK [4] and CPS-1 [5] when provided protein supplements, while others have not observed changes on hepatic mRNA expression of expression of PEPCK [4] and CPS-1 [6] when provided energy and protein supplements, respectively.

CPS-1 is expressed in hepatocytes and epithelial cells of the intestinal mucosa and is considered a rate-limiting step within the urea cycle by converting ammonia into carbamoyl phosphate. In addition, the expression and activity of urea cycle enzymes are affected by hormones and nutrients [5]. PEPCK, on the other hand, is a potentially rate-limiting enzyme for hepatic gluconeogenesis from precursors that enter the gluconeogenic path before the triose phosphates. Thus, PEPCK catalyses the conversion of oxaloacetate to phosphoenolpyruvate [7].

Hence, we hypothesised that a protein-rich supplement would improve nutritional and productive performance, as well as metabolic responses in grazing suckling female beef calves. Thus, this study was conducted to evaluate the effects of the provision of a protein-rich supplement on nutritional and productive performance and metabolic profile, as well as the gene expression of CPS-1 and PEPCK on grazing suckling female beef calves in tropical conditions.

## MATERIALS AND METHODS

### Animal care

This study was approved by the Brazilian Ethics Committee on Animal Use (CEUAP/UFV – process no. 18/2018), according to ethical principles of animal experimentation established by the National Council of Animal Experimentation Control (CONCEA).

### Location and weather conditions

The experiment was performed at the Beef Cattle Farm of the Animal Science Department of Federal University of Viçosa, Viçosa-MG, Brazil, from January to June 2016 (150 d of experimentation), which corresponded to the rainy season and the rainy-dry transition season. Rainfall summed to 598 mm during the experiment and the average temperature was 21.4°C.

### Animals, diets, and experimental design

Fifty-six Nellore suckling female calves (2.7±0.1 mo age) with an average initial body weight [BW] of 115±2.3 kg were used. The BW of their respective dams was 490±8.5 kg. The animals underwent 14 d of adaptation to the diet and experimental paddock area before the evaluation phase.

The cow-calf units were kept in eight 7 hectares paddocks each (seven cow-calf units in each paddock and four paddocks for each treatment), which were evenly formed by *Brachiaria decumbens* Stapf. and equipped with drinkers and feeders. All animals had free access to water and a mineral mixture (500 g/kg CaHPO_4_; 471.9 g/kg NaCl; 15 g/kg ZnSO_4_; 7 g/kg Cu_2_SO_4_; 500 mg/kg CoSO_4_; 500 mg/kg KIO_3_; 100 mg/kg Na_2_SeO_3_, and 5 g/kg MnSO_4_).

The animals were distributed in a completely randomised design and made to undergo two treatments as follows: UNS (without supplementation), and SUP (supplementation with 5 g/kg BW of a protein supplement). The supplement was delivered daily at 10:00 h in the creep-feeding system and composed of soybean meal, corn meal and wheat meal and formulated to contain 30% of crude protein (CP) as fed. The chemical compositions of the protein supplement and pasture are shown in Table 1.

The supplementation level of 5 g/kg BW corresponded to approximately 45% and 23% of the dietary requirements of CP and energy, respectively, for young female Zebu (5 to 6 mo of age) under grazing conditions with BW of 200 kg and expected gain of 1 kg/d [1].

Female calves were weighed at 06:00 h every 30 d (without fasting) to adjust the amount of supplement to be provided.

### Sampling and chemical analysis

Representative samples of supplement were collected monthly. Pasture chemical composition was assessed by hand-plucked samples every 15 d, based on the identification of the places of intake and the parts of the plant selected by the animals, simulating the female calves’ grazing as closely as possible. Additionally, a second pasture sample was collected every 15 d to estimate the forage’s potentially digestible dry matter (pdDM), as recommended by Detmann et al [8]. The area to be sampled was delimited by a 0.5×0.5-m metal frame in four sites chosen randomly in each experimental paddock and cut at approximately 1 cm above the soil. The samples of supplement and pasture were identified, weighed and oven-dried in a forced-air circulation oven immediately after collection (Ar SL – 102; SOLAB, Piracicaba, SP, Brazil) at 60°C for 72 h, and subsequently grounded with a 1- and 2-mm knife Willye mill (TE-680; SOLAB, Brazil) and stored in plastic pots before analysis.

The dams were milked on d 25, 75, and 125 of the experiment to estimate the milk yield converted to a 24-h and composition of daily milk intake by the female calves, following the procedures described by Almeida et al [9]. From the milk obtained on the collection days, the average milk production of the dams during the experiment was calculated. Additionally, the milk obtained on the 75th d was used to estimate intake in the digestion trail and analysed for lactose, protein, fat, and total solids content using an infrared spectrophotometer (Foss MilkoScan FT120, Hillerød, Denmark). Dam milk produced was corrected to 4% of fat (Milk_4%_) calculated by the following equation [10]:


$${\text{Milk}}_{4\%}\;(\text{kg}/\text{d})=0.4\times (\text{milk production kg}/\text{d})+[15(\text{fat production g}/\text{kg}\times \text{milk production kg}/\text{d}/100)]$$

A 9-d intake and digestion trial was carried out from d 75 of the experiment to evaluate the nutrient intake and digestibility of calves. Chromium oxide (Cr_2_O_3_), in the amount of 10 g/animal/d, was used as an external marker to estimate faecal excretion. The Cr_2_O_3_ was packed in paper cartridges and delivered via the esophagus with a metal probe once daily at 10:00 h over 8 d. Individual intake of the supplement was estimated using titanium dioxide (TiO_2_) mixed in the supplement, in a plastic bag, in a proportion of 10 g/kg of supplement for 8 d. Finally, indigestible neutral detergent fibre (iNDF) was used as an internal marker to estimate dry matter (DM) intake. Six days were used for the stabilisation of marker excretion, and faecal samples were subsequently collected immediately after defecation or directly from the rectum of the animals in amounts of approximately 200 g at 18:00, 16:00, 10:00, and 06:00 h on d 6, 7, 8, and 9 of the digestion trial, respectively. All the faecal samples were oven-dried (60°C for 72 h) and ground as described for pasture. Grounded samples were pooled over the sampling time points by calf and stored in plastic pots before analysis.

Samples of supplement, forage, and faeces were analysed for DM (dried overnight at 105°C; method INCT-CA number G-003/1), ash (complete combustion in a muffle furnace at 600°C for 4 h; method INCT-CA number M-001/1), nitrogen (Kjeldahl procedure; method INCT-CA number N-001/1), ether extract (Randall procedure; method INCT-CA number G-005/1), neutral detergent fibre (NDF; method INCT-CA number-002/1), NDF corrected for ash (neutral detergent insoluble ash [NDIA]; method INCT-CA number M-002/1) and protein (neutral detergent insoluble protein [NDIP]; method INCT-CA number N-004/1) residue (NDFap; using a heat-stable α-amylase, omitting sodium sulfite and correcting for residual ash and protein), according to the standard analytical procedures of the Brazilian National Institute of Science and Technology in Animal Science (INCT-CA) [11].

The NDFap was quantified using the following equation [11]:


NDFap=NDF×[(100-NDIP-NDIA)/100]

The content of iNDF in samples of faeces, forages, and supplement (ground through 2-mm sieves) was estimated as the residual NDF remaining after 288 h of *in situ* ruminal incubation using F57 filter bags (Ankom Technology Corp., Macedon, NY, USA), according to Detmann et al [11].

The non-fibrous carbohydrates (NFC) were quantified, according to Detmann et al [11]:


NFC=100-MM-EE-NDFap-CP

where MM = mineral matter; EE = ether extract; NDFap = neutral detergent fibre corrected for ash and protein residue; CP = crude protein.

Faecal samples were also analysed for chromium concentration using nitroperchloric digestion and atomic absorption spectrophotometry and titanium dioxide by colorimetry [11].

The pdDM in forage available on pasture was estimated using the following equation [8]:


pdDM=0.98×(100-NDF)+(NDF-iNDF)

The faecal DM excretion was estimated using the chromic oxide marker, based on the ratio between the amount of chromium supplied and its concentration in the faeces [8]:


Faecal DM (kg/d)=AOI/ICF

where AOI = amount of indicator ingested (g); ICF = indicator concentration in faecal DM (g/kg of faecal DM).

Individual supplement intake (ISI) was estimated by the relation of excretion of TiO2 in faeces and marker concentration in the supplement as follows [8]:


ISI=[(FE×ICaF)/IOG]×SOG

where ISI = individual supplement intake (kg/d); FE = faecal DM excretion (kg/d); ICaF = indicator concentration in animal faeces (kg/kg); IOG = indicator present in the supplement offered to each group (kg/d); SOG = supplement amount offered to the group of animals or treatment (kg/d).

Individual DM intake (DMI) was estimated by using iNDF as an internal marker and calculated by the following equation [8]:


DMI=[(FE×iNDFF-iNDFS)/iNDFP]+ISI+MI

where FE = faecal DM excretion (kg/d); iNDFF = concentration of iNDF in the faeces (kg/kg); iNDFS = concentration of iNDF in the supplement (kg/kg); iNDFP = amount of iNDF form pasture (kg/kg); ISI = individual supplement intake (kg/d); and MI = milk intake (kg/d).

To evaluate the MICN and urinary urea nitrogen (UUN) excretion, spot urinary samples were collected during spontaneous urination on the last day of the digestion trial, 4 h before and after the provision of the supplement. After the collection, 10 mL of urine were diluted in 40 mL of H_2_SO_4_ (0.036 N) and frozen at −20°C for later analysis.

Urine was analysed for creatinine (K067; Bioclin Quibasa, Belo Horizonte, Brazil) quantified by kinetic colorimetric method, urea (K056; Bioclin Quibasa, Brazil) and uric acid (K0139, Bioclin Quibasa, Brazil) by enzymatic-colorimetric methods and allantoin as recommended by Chen and Gomes [12].

Ruminal synthesis of nitrogen compounds was calculated using the equation described by Barbosa et al [13]:


MICN=(70×AP)/(0.93×0.137×1,000)

where MICN = microbial nitrogen production (g/d); AP = absorbed purines (mmol/d); 70 = purine N content (mg/mol); 0.93 = purine digestibility and 0.137 = relation of purine N:total N of microorganisms.

### Blood samples and measurements

On d 45, 90, and 135, blood was collected to quantify the serum concentration of urea nitrogen, total proteins, albumin, globulins, and triglycerides, as well as the plasma concentration of glucose. On d 135, a blood sample was collected to quantify the serum insulin-like growth factor-1 (IGF-1) concentration. All samples were collected at 07:00 h via jugular venipuncture in vacuum tubes with clot activator and gel for serum separation (BD Vacutainer SST II Advance, São Paulo, Brazil) and vacuum tubes containing sodium fluoride and EDTA (BD Vacutainer Fluoreto/EDTA, São Paulo, Brazil) as glycolytic inhibitor and anticoagulant, respectively, for plasma preparation. Immediately after collection, samples were centrifuged (3,600×g for 20 min) and serum and plasma were frozen at −20°C for later analysis.

The IGF-1 was analysed by chemiluminescence using Liaison reagent (313231; DiaSorin, Vercelli, Italy) in the Liaison analyser (DiaSorin, Italy). Glucose (K082; Bioclin Quibasa, Belo Horizonte, Brazil), triglycerides (K117, K082; Bioclin Quibasa, Brazil) and urea (K056, K082; Bioclin Quibasa, Brazil) were quantified by enzymatic-colorimetric methods, and total proteins (K031, K082; Bioclin Quibasa, Brazil) and albumin (K040, K082; Bioclin Quibasa, Brazil) by colorimetric methods. Globulins concentrations were calculated as the difference between the analysed total protein and albumin content. Serum urea nitrogen (SUN) was estimated as 46.67% of the total serum urea. All blood parameters were analysed following the manufacturer’s instructions in an automatic biochemistry analyser (BS200E; Mindray, Shenzhen, China).

### Hepatic tissue biopsy and mRNA gene expression

Twelve animals from each treatment were randomly selected at the end of the experiment for hepatic tissue biopsies. Liver sampling was performed with a Tru-Cut 3-mm diameter needle (PJT1115, 11G x 15 cm notch; MDL SRL, Delebio, Italy) at 07:00 h on d 145 of the experimental period, according to procedures described by Mølgaard et al [14]. The incision was made between the 11th and 12th ribs for the collection of samples from the right hepatic lobe [15]. Liver samples (approximately 200 mg of tissue) were immediately placed in cryotubes, frozen and stored in liquid nitrogen at −196°C until processing.

Total RNA extraction was performed from 50 mg of liver samples using RNeasy Mini Kit (Qiagen, Valencia, CA, USA). The RNA integrity (RIN) was evaluated by capillary electrophoresis using an RNA 6000 Nano kit and 2100 Bioanalyser System (Agilent Techonologies, Santa Clara, CA, USA). Samples with RIN >7.0 were, subsequently, treated with DNAse I Amplification Grade (Invitrogen, Waltham, MA, USA) and RNA concentration was estimated by NanoVue Plus spectrophotometer (GE Healthcare, Freiburg, Germany). The first strand of cDNA synthesis was performed using a GoScript Reverse Transcriptase kit (Promega, Madison, WI, USA). Samples were stored at −20°C for later analysis. Primers for target gene amplification and endogenous amplification were designed using PrimerQuest program with sequences obtained from Gen-Bank database, being: CPS-1, sense 5’-ACA CTG GCT GCA GAA TAC CC-3’ and antisense 5’-TTC TTG CCA AGC TGA CGC AA-3’; PEPCK, sense 5’-CCC CCA GAG ATC AAG AAT CA-3’ and antisense 5’-ATT GGA GGT GGA CAG TCA GG-3’). The 18S ribosomal RNA (18S; NR_036642.1) was used as the endogenous control gene. Serial dilution of cDNA was used to determine the amplification efficiency and optimal primer concentration for each gene. Quantitative real-time polymerase chain reaction (qPCR) reactions were performed in an ABI Prism 7300 Sequence Detection Systems thermocycler (Applied Biosystems, Foster City, CA, USA) using a GoTaq qPCR Master Mix (Promega Corporation, USA). The qPCR reaction consisted of the following three cycle parameters: 95°C for 3 min, 40 cycles at 95°C for 10 s, and 60°C for 30 s, followed by melting curve analysis (0.01°C/s) to confirm product purity. Results were expressed relative to 18S using the ΔΔCt method [16].

### Productive performance, body measures, and carcass characteristics

For productive performance evaluation, all calves were weighed on d 1 and 150 of the experiment phase after 14 h for solids and milk fasting, allowing only water intake. Weighing at 150 d coincided with weaning at 7.7±0.1 mo of age.

On d 150 in the experimental period, body measures were taken to evaluate the body growth of the animals. The rump width (the maximum distance between iliac tuberosities), rump length (from the ischial tuberosity to the iliac tuberosity), rib depth (vertically from the highest point over the scapulae to the endpoint of the rib), body length (from the anterior point of the scapulae vertically to the posterior midline), height at withers (from the highest point of the shoulder blade to the ground) and rump height (from the iliac tuberosity vertically to the ground) were measured with a height stick (cm) and recorded. The heart girth (the body circumference immediately posterior to the front legs) was measured with a flexible tape. Concurrently, carcass characteristics were evaluated by ultrasound (Aloka SSD 500; 3.5-MHz linear probe; Aloka Co, Tokyo, Japan). Carcass images were obtained between the 12th and 13th ribs over the longissimus muscle to measure the ribeye area and backfat-thickness on longissimus muscle (FAT-Ld), and between the ischium and pubis to measure the backfat-thickness on rump (FAT-R). Vegetable oil was used to ensure adequate acoustic contact. Images were analysed using the Bio Soft Toolbox II for Beef software (Biotronics Inc., Ames, IA, USA).

### Statistical analysis

The experiment was conducted and analysed in a completely randomised design with a duple error structure. The MIXED procedure of the SAS 9.4 software was used for all statistical analyses. The effect of treatment on all variables measured was evaluated by analysis of variance, according to the following mathematical model:


$${\text{Y}}_{\text{ijk}}=\mu +Ti\;(0,1)+{\text{e}}_{(\text{i})\text{j}}+{ɛ}_{(\text{ij})\text{k}}$$

where, Y_ijk_ = observations of individual k on paddock j under treatment i; μ = overall mean; *Ti* = fixed effect of treatment, being 0, 1 = UNS, female calves without supplementation; SUP, female calves supplementation with 5 g/kg BW of protein supplement; e_(i)j_ = random error, unobservable, associate to each j paddock under treatment i, assumed to be normally and independently distributed (NID; 0, σe^2^); and ɛ_(ij)k_: random error, unobservable, associate to each k observation on j paddock under treatment i, assumed to be NID (0, σe^2^).

The blood concentrations, except IGF-1, were analysed as time-repeated measures where day of collection was considered the repeated variable. The choice of the most appropriate covariance structure was based on the lowest value of the corrected Akaike information criterion. Differences were considered significant at p≤0.05.

## RESULTS

### Intake and nutritional characteristics

In this study, the average availability of total DM and pdDM of the forage on pasture were 4.5±0.17 and 3.0±0.14 t/ha, respectively. The forage samples collected by the hand-plucked method and protein supplement had an average CP, NFC, EE, and NDFap content of 7.83%, 20.3%, 1.5%, and 61.6% of the forage DM and 30.0%, 40.1%, 1.6%, and 20.0% of the DM of protein supplement, respectively (Table 1).

Milk yield and composition of the dams were not affected by the provision of the protein supplement to their female calves (Table 2). Furthermore, the milk intake in the female calves did not differ during the experimental period (Table 3). However, SUP female calves showed a higher (p≤0.03) voluntary intake of total DM, forage DM, organic matter (OM), CP, NDFap, NFC, digested OM (DOM), a higher CP:DOM ratio, TDN and ME (Table 3).

There was an increase (p≤0.03) in the digestibility of OM, CP and DOM in SUP female calves compared to UNS female calves, while the digestibility of NDFap and NFC was not affected by treatment. Microbial N production and UUN increased in response to the protein supplement (p≤0.02). The ratio of microbial N produced relative to ingested N (MICNR) and the efficiency of microbial protein synthesis (EMS) were not affected by supplementation (Table 3).

### Metabolic profile

**Figure 1:**
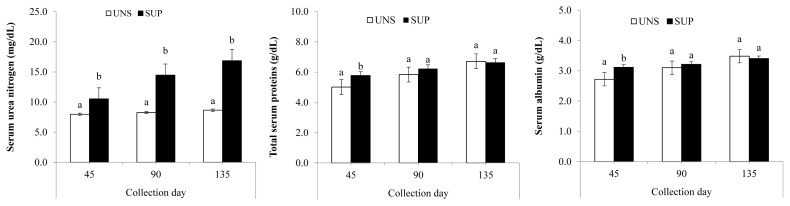
Serum concentration of urea nitrogen, total proteins, and albumin in grazing suckling female beef calves fed a protein supplement. UNS, without supplementation; SUP, supplementation with 5 g/kg body weight of protein supplement. ^a,b^ Means without a common lowercase letter differ significantly (p≤0.05).

Interaction (p≤0.03) was observed between treatments and collection days for SUN, total proteins, and albumin. SUP female calves showed a higher SUN level throughout the experiment (p≤0.01). SUP female calves had higher total protein and albumin compared to UNS female calves at d 45 (p≤0.04), but there was no difference in their concentrations between the treatment groups at d 90 or 135 (Figure 1). SUP female calves displayed higher (p≤0.03) serum IGF-1 concentrations than UNS female calves, with detected in the blood of supplemented animals. Blood glucose, triglyceride and globulins levels were not affected by supplementation (Table 4).

**Figure 2:**
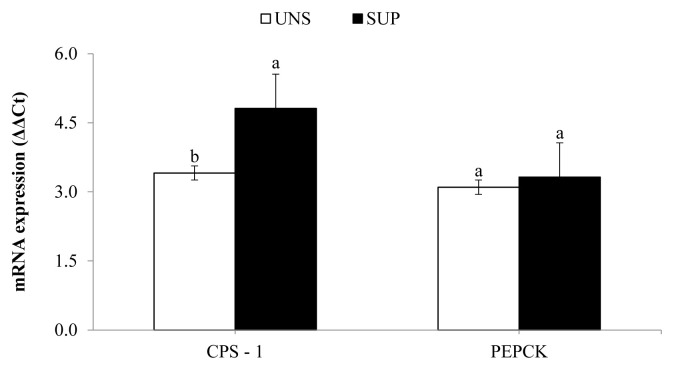
Hepatic mRNA expression of carbamoyl phosphate synthetase-1 (CPS-1) and phosphoenolpyruvate carboxykinase (PEPCK) in response to a protein supplement for grazing suckling female beef calves as measured by quantitative real-time polymerase chain reaction. UNS, without supplementation; SUP, supplementation with 5 g/kg body weight of protein supplement. ^a,b^ Means without a common lowercase letter differ significantly (p≤0.05).

Hepatic mRNA expression of CPS-1 increased (p≤0.04) in SUP female calves relative to UNS female calves, while the expression of PEPCK was not affected by supplementation (Figure 2).

### Performance, body measures, and carcass characteristics

The final BW (FBW) and average daily gain (ADG) from SUP female calves were higher (p≤0.02; Table 5) compared with UNS female calves. Supplementation did not affect the ribeye area, FAT-Ld, or FAT-R. In general, the protein supplement did not affect the body growth of the female calves.

## DISCUSSION

The forage grazed in this experiment had a mean CP content (<8%) below the critical limit to maintain rumen microbial growth in support of fibre digestion [17]; consequently, this ruminal nitrogen deficiency was the main constraint for maximisation of voluntary intake and digestibility of the pasture ingested by the female calves [3]. Under these circumstances, the provision of protein supplement can increase CP intake to levels that will optimise fibre digestion and allow greater intake of low CP forage by grazing female calves [3,18].

In addition, the voluntary intake of forage is maximised with a 216 g CP/kg DOM ratio in the diet [19]. In this regard, our results showed a higher CP:DOM ratio in SUP female calves when compared to UNS female calves (229 vs 198 g CP/kg DOM). Thus, SUP female calves had values above and closer to those recommended to favour a higher forage intake and degradation, suggesting a better equilibrium of protein to energy ratio. This metabolic adequacy in the SUP female calves can explain the higher intake not only in total DM but also of forage DM and nutrients, as also reported in the studies of Da Silva et al [20] and Ortega et al [21].

Furthermore, the increase in OM, CP, and DOM intake in the SUP female calves is due to the intake of supplements and its positive effect on the degradation of these components in the diet.

On the other hand, despite the higher NFC intake in SUP female calves compared to UNS female calves, there was no effect on the NFC digestibility coefficient, which may be associated with the high participation of milk (high digestible components) in the total diet. In addition, Detmann et al [22] suggested that the pattern was related to both the high and constant digestibility of NFC, guaranteeing the status of a nutritional entity.

The increase in the SUP female calves’ MICN is associated with the supplement offer because of the higher supply of protein and energy substrates for the growth of the ruminal microorganisms [21]. Likewise, our data revealed the supplementation increased urine urea nitrogen excretion. This response suggests that there is a lower percentage of ingested nitrogen directed to recycling and a greater percentage of nitrogen available for production.

The pattern observed for UUN can be indicative of a fraction of N, whose efficiency of use can be optimised and lead to an increase in the EMS. Therefore, better synchrony of the ratio of protein to energy would be necessary. These results are consistent with those reported by Ortega et al [21].

On the other hand, the increase in the mRNA expression of CPS-1 suggests that ammonia absorption across the rumen wall was higher in SUP female calves, due to higher intake and degradation of protein in the rumen. This response would be related to an increase in hepatic urea synthesis and serum urea nitrogen concentration, as well as the urine urea N excretion, as observed in this experiment [23].

According to Batista et al [24], two aspects can be considered regarding nitrogen usage. First, the smaller amount of absorbed N would be used to supply ruminal ammonia N, increasing the use of N for protein synthesis, which perhaps could lead to an increase in performance or UUN excretion in SUP female calves. Second, lower nitrogen intake of cattle in tropical conditions would result in a higher ruminal N demand and tend to decrease their urinary N excretion and increase the proportion of N recycled to the rumen, which perhaps led to the lower rates of gain and explains the decreasing pattern in performance or UUN excretion from UNS female calves.

Regarding the total protein and albumin concentrations observed, the lower levels of these metabolites at 45 experimental days (4.2 mo of calves’ age) in female calves, without access to the supplement, suggest lower nutritional status. This fact is possibly due to the greater dependence on the dam’s milk and less ruminal capacity to degrade fibre at that time. By contrast, Da Silva et al [20] did not report an increase in the serum levels of total proteins, albumins, or globulins in female calves with or without supplementation.

The provision of supplement increased the serum concentration of IGF-1 in SUP female calves, indicating that the effects of higher CP (nitrogen) and DM were essentially anabolic, this is consistent with higher BW and ADG during the experimental period. The IGF-1 is an endocrine regulator of muscle growth in cattle. In addition to its independent action, it promotes important links between growth hormone and the metabolic growth process, particularly in skeletal muscle [25,26]. The IGF-1 concentrations are responsive to nutritional plane as well as the diet composition, with an apparently more prominent response to dietary protein concentration than energy concentration [26]. This justifies the increase of its serum concentration with the protein supply. Similar results have been obtained by Rodríguez-Sánchez et al [27] and Franco et al [28], who supplemented cattle in tropical conditions with protein supplements.

Despite this, the higher IGF-1 concentrations were not sufficient to influence the ribeye area, FAT-Ld or FAT-R, indicators of muscle and adipose tissue deposition. Such a pattern seems to indicate that both the anabolic effect and the level of protein supplement offered were not sufficient to influence the gain composition. Da Silva et al [20] and Ortega et al [21] did not observe an increase in ADG, FBW or ribeye area, although they reported an increase in FAT-R and a tendency to increase in FAT-Ld with an increase in the supplement amount.

From another perspective, despite the higher nutrient intake in SUP female calves compared to UNS female calves, there were no differences during the experiment in the gene expression of PEPCK mRNA, a key enzyme for hepatic gluconeogenesis. These results are consistent with blood glucose and triglyceride concentrations observed in the experimental animals. In this context, the similar energy status observed among treatments may have limited the deposition of bodily tissues [20,29].

The lack of effect on the height at the withers and the rump indicates that the supplementation tested did not influence the skeletal development of the animals, since these measurements are mainly composed of the measurement of the long bones in the animal and are good indicators of body growth [27]. In contrast, some authors reported differences in height due to the nutritional treatment applied [27,30].

The absence of differences in heart girth, rump width and length, rib width and body length indicates that the treatments applied promoted adequate tissue development in animals since both the gain rates and metabolic profiles observed are consistent with the coexistence of an anabolic condition [21]. These variables are associated with the prediction of animals’ BW, food intake capacity, distribution of prime cuts in the hindquarter, internal pelvic area and the incidence and difficulty in calving in primiparous heifers [27].

In summary, provision of a protein-rich supplement improves the intake and nutrients digestibility, average daily gain and final BW and increases metabolic indicators of the protein status, as well as increases CPS-1 mRNA expression in grazing suckling female beef calves in tropical conditions.

## IMPLICATIONS

Cattle under grazing in tropical conditions are fed a forage-based diet. However, tropical grasses show qualitative and quantitative variability throughout the year, which can limit the animals’ growth rates. The supplementation in the creep-feeding system has been used to correct metabolic unbalances generated by the characteristics of the ingested diet and optimise the performance of grazing suckling beef calves. Our results showed that protein supplementation improves forage intake, nutrients digestibility, BW daily gain and metabolic responses in grazing suckling female beef calves in tropical conditions.

## Figures and Tables

**Table 1:** Nutrient composition of protein supplement and forage during the experiment

Item	Protein supplement	Forage^1)^
Ingredient % (as-fed basis)		
Soybean meal	50.0	-
Corn meal	30.0	-
Wheat meal	20.0	-
Chemical composition (% of DM)		
Dry matter (% fresh matter)	89.3	88.9±0.77
Organic matter	91.7	91.3±2.92
Crude protein	30.0	7.8±0.37
Ether extract	1.6	1.5±0.26
Non-fibrous carbohydrates	40.1	20.3±0.61
NDFap	20.0	61.6±0.55
iNDF	2.7	14.5±0.73

DM, dry matter; NDFap, neutral detergent fibre corrected for ash and protein residue; iNDF, indigestible neutral detergent fibre.

1) Mean±standard error of the mean of chemical composition of forage during the experiment.

**Table 2:** Milk yield, fat-corrected milk yield, and milk composition of cows according to the treatment applied to their female beef calves during the experiment

Item	Treatments^1)^		SEM	p-value

	UNS	SUP		
Milk (kg/d)	5.71	5.88	0.294	0.70
Milk_4%_ (kg/d)^2)^	6.83	6.9	0.296	0.87
Fat (%)	5.34	5.23	0.15	0.62
Protein (%)	3.52	3.46	0.04	0.44
Lactose (%)	4.56	4.51	0.04	0.34
Total solids (%)	12.42	14.29	0.16	0.61

SEM, standard error of the mean.

1) UNS, without supplementation; SUP, supplementation with 5 g/kg BW of protein supplement.

2) Milk_4%_ = milk production corrected to 4% of fat; Milk_4%_= 0.4×(milk production kg/d)+[15 (fat production g/kg× milk production kg/d/100)].

Values differ significantly at p≤0.05.

**Table 3:** Voluntary intake (kg/d), nutrient apparent digestibility coefficients (g/g), and nitrogen synthesis and excretion in grazing suckling female beef calves fed a protein supplement

Item	Treatments^1)^		SEM	p-value

	UNS	SUP		
Intake				
Total DM^2)^	3.07	4.46	0.166	<0.01
Forage DM	2.08	2.55	0.128	0.03
Milk	0.99	0.99	0.044	0.98
Organic matter	2.85	4.14	0.146	<0.01
Crude protein	0.41	0.72	0.018	<0.01
NDFap	1.27	1.78	0.069	<0.01
Non-fibrous carbohydrates	0.77	1.22	0.046	<0.01
Digested organic matter	2.09	3.14	0.103	<0.01
CP:DOM^3)^	198	229	3.0	<0.01
TDN	2.52	3.60	0.123	<0.01
ME (Mcal/kg)	8.98	12.81	0.4412	<0.01
Digestibility				
Organic matter	0.734	0.758	0.0055	0.02
Crude protein	0.724	0.784	0.0097	<0.01
NDFap	0.613	0.622	0.0148	0.67
Non-fibrous carbohydrates	0.863	0.899	0.0183	0.21
Digested organic matter (g/kg DM)	682	703	5.6	0.03
Nitrogen synthesis and excretion				
Microbial N production (g/d)	41.5	66.2	5.93	0.02
MICNR (g/g N)	0.653	0.595	0.0682	0.56
EMS (g/kg DOM)	126.8	133.6	14.04	0.74
Urine urea N excretion (g/d)	21.9	39.6	2.24	<0.01

SEM, standard error of the mean; DM, dry matter; NDFap, neutral detergent fibre corrected for ash and protein residue; TDN, total digestible nutrients; ME, metabolizable energy; MICNR, ratio of microbial N produced relative to ingested N from total DM; EMS, efficiency of microbial protein synthesis.

1) UNS, without supplementation; SUP, supplementation with 5 g/kg BW of protein supplement.

2) Total DM, sum of DM intake from forage, milk, and protein supplement.

3) CP:DOM, ratio of crude protein to digested organic matter.

**Table 4:** Blood concentrations of IGF-1, serum proteins, and metabolites in grazing suckling female beef calves fed a protein supplement

Item	Treatments^1)^		SEM	p-value^2)^	
	
	UNS	SUP		TREAT	TREAT×COL
IGF-1 (ng/mL)	225.1	289.3	16.31	0.03	-
SUN (mg/dL)	8.3	14.0	0.41	<0.01	<0.01
Total proteins (g/dL)	5.86	6.22	0.105	0.01	0.03
Albumin (g/dL)	3.1	3.24	0.058	0.10	<0.01
Globulins (g/dL)	2.76	2.97	0.075	0.07	0.25
Glucose (mg/dL)	87.5	87.6	3.18	0.97	0.48
Triglycerides (mg/dL)	33.7	32.7	1.91	0.72	0.31

IGF-1, insulin-like growth factor-1; SEM, standard error of the mean; SUN, serum urea nitrogen.

1) UNS, without supplementation; SUP, supplementation with 5 g/kg BW of protein supplement.

2) TREAT = treatment effect; TREAT×COL = treatment×collection day interaction effect.

**Table 5:** Productive performance, carcass characteristics, and body development in grazing suckling female beef calves fed a protein supplement

Item	Treatments^1)^		SEM	p-value

	UNS	SUP		
Initial BW (kg)	115.4	115.4	3.33	0.99
Final BW (kg)	225.4	247.3	5.16	0.02
Average daily gain (g/d)	734	880	34.4	0.02
Ribeye area (cm^2^)	39.3	41.5	1.03	0.18
FAT-*Ld* (mm)	1.98	2.04	0.161	0.81
FAT-*R* (mm)	2.8	3.08	0.222	0.40
Height at withers (cm)	116.9	118.5	0.64	0.11
Heart girth (cm)	136.1	143.2	2.85	0.12
Rib width (cm)	38.1	38.4	0.46	0.59
Rump height (cm)	124.5	125.9	0.62	0.15
Rump width (cm)	35.3	35.3	0.37	0.97
Rump length (cm)	40.1	40.1	0.32	0.94
Body length (cm)	118.4	120.9	1.00	0.12

SEM, standard error of the mean; BW, body weight; FAT-Ld, backfat-thickness on longissimus muscle; FAT-R, backfat-thickness on rump.

1) UNS, without supplementation; SUP, supplementation with 5 g/kg BW of protein supplement.
